# Antimicrobial Use, Residues, Resistance and Governance in the Food and Agriculture Sectors, Tanzania

**DOI:** 10.3390/antibiotics10040454

**Published:** 2021-04-16

**Authors:** Robinson H. Mdegela, Elibariki R. Mwakapeje, Bachana Rubegwa, Daniel T. Gebeyehu, Solange Niyigena, Victoria Msambichaka, Hezron E. Nonga, Nicolas Antoine-Moussiaux, Folorunso O. Fasina

**Affiliations:** 1 Department of Veterinary Medicine and Public Health, Sokoine University of Agriculture, Morogoro 3006, Tanzania; mdegegela@sua.ac.tz; 2 Emergency Centre for Transboundary Animal Diseases (ECTAD), Food and Agriculture Organization of the United Nations (FAO), Dar es Salaam 14111, Tanzania; elibariki.mwakapeje@fao.org (E.R.M.); BRubegwa@path.org (B.R.); msambichakavictoria@yahoo.com (V.M.); 3 Faculty of Veterinary Medicine, University of Liège, 4032 Liege, Belgium; dteshome11@gmail.com (D.T.G.); niso20052000@yahoo.fr (S.N.); nantoine@uliege.be (N.A.-M.); 4 Department of Veterinary Medicine, School of Veterinary Medicine, Wollo University, Dessie 6000, Ethiopia; 5 Directorate of Veterinary Services, Ministry of Livestock and Fisheries, Dodoma 41000, Tanzania; nongahezron@yahoo.co.uk; 6 Department of Veterinary Tropical Diseases, Faculty of Veterinary Science, University of Pretoria, Onderstepoort 0110, South Africa

**Keywords:** antibiotic use, antibiotic residues, antibiotic resistance, food systems, developing countries

## Abstract

All infections are potentially curable as long as the etiological agents are susceptible to antimicrobials. The increased rate at which antimicrobials are becoming ineffective is a global health risk of increasing concern that threatens withdrawal of beneficial antimicrobials for disease control. The increased demand for food of animal origin, in particular eggs, meat and milk has led to intensification and commercial production systems where excessive use and misuse of antimicrobials may prevail. Antimicrobials, handled and used by farmers and animal attendants with no formal education, may be predisposed to incorrect dosages, misuse, incorrect applications and non-adherence to withdrawal periods. This study was conducted to assess the regulatory roles and governance of antimicrobials, establish the pattern and extent of their use, evaluate the antimicrobial residues and resistance in the food animals and crop agriculture value chains, and relate these findings to existing strategies in place for combating the emergence of antimicrobial resistance in Tanzania. A multimethod approach (desk review, field study and interviews) was used. Relevant establishments were also visited. High levels of resistance to penicillin G, chloramphenicol, streptomycin and oxytetracycline have been reported, especially for *Actinobacter pyogenes*, *Staphylococcus hyicus*, *Staphylococcus intermedius* and *Staphylococcus aureus* from dairy cattle with mastitis and in humans. Similar trends were found in poultry where eggs and meat are contaminated with *Escherichia coli* strains resistant to amoxicillin + clavulanate, sulphamethoxazole and neomycin. An increasing trend of emerging multidrug resistant *E. coli*, *Klebsiella pneumoniae*, *Staphylococcus aureus* and *Salmonella* was also found in food animals. An increase in methicillin resistant *Staphlococcus aureus* (MRSA) and extended-spectrum beta-lactamase (ESBL) in the livestock sector in Tanzania have been reported. The pathogens isolated in animals were resistant to ampicillin, augmentin, gentamicin, co-trimoxazole, tetracycline, amoxicillin, streptomycin, nalidixic acid, azithromycin, chloramphenicol, tylosin, erythromycin, cefuroxime, norfloxacin and ciprofloxacin. An increased usage of antimicrobials for prophylaxis, and therapeutics against pathogens and for growth promotion in livestock, aquaculture and crop production were observed. A One Health strategic approach is advocated to combat antimicrobial resistance (AMR) in the food and agriculture sectors in Tanzania. Practical recommendations include (a) legislation review and implementation; (b) antimicrobial use (AMU), AMR and antimicrobial residue (AR) awareness and advocacy among stakeholders along the value chain; (c) strengthening of surveillance and monitoring programs for AMU, AMR and AR; (d) enhanced development and use of rapid and innovative diagnostic tests and the promotion of biosecurity principles; and (e) good husbandry practices. The utilization of this information to improve public health policies and reduce the burden of AMR will be beneficial.

## 1. Introduction

Agriculture is Tanzania’s economy mainstay, contributing nearly to 30% of the country’s gross domestic product (GDP) and 67% of total employment. Tanzania has the third largest livestock population on the African continent. The Tanzania Livestock Master Plan (2017/2018–2021/2022) revealed that 50% of households keep livestock (4.493 million households), 62% of which are rural and 23% urban-based. The ownership patterns in terms of dominance rank chickens as the highest (86% of the households), followed by goats (48%), cattle (35%), pigs (9%) and other livestock (10%). Meat production in Tanzania is estimated at about 662,931 metric tonnes for the year 2020 to 2022 [1].

In 2015, Tanzania’s Ministry of Livestock and Fisheries launched the Tanzania Livestock Modernization Initiative (TLMI), and recently, in 2019, the Tanzania Livestock Master Plan (TLMP) was launched to transform the traditional livestock sub-sector into a modern, responsive, sustainable and environmentally friendly engine for rural development.

However, livestock diseases hinder the development of this sector and expose producers to high livelihood risks and uncertainties [2]. The intensification of livestock farming, large number of wildlife and close interaction of humans and animals aggravate the circulation of zoonotic and infectious diseases in animal and human populations and equally affects the food chain in Tanzania [3]. In addition to the challenge of emerging and zoonotic diseases, increasing intensification and mass production of animals also expels the poor rural farmers from the market [4].

Antimicrobials are agents that kill microorganisms or suppresses their multiplication or growth [5], and the failure of antimicrobials’ effect against the growth and multiplication of microorganisms is called antimicrobial resistance (AMR). AMR is a mechanism that evolves in microbes to resist the bactericidal/bacteriostatic activity of antimicrobials. This happens when microorganisms (such as bacteria, fungi, viruses and parasites) change the typical response patterns when they are exposed to antimicrobials (such as antibiotics, antifungals, antivirals, antimalarials and anthelmintics) [6].

Although different factors may aggravate AMR, over-use and misuse of antimicrobials in food and agriculture production play a major role in the emergence and spread of AMR. Indeed, AMR emergence and diffusion correspond to a selective process that allows bacterial populations to adapt to their environment. Hence, AMR is inextricably tied to antimicrobial use (AMU) and will be favored when this use is sub-optimal, abused or widespread. In particular, avoidable practices that are recognised as key contributors to AMR are AMU in animal production for growth promotion, prophylaxis and metaphylaxis and AMU without professional oversight or after poor diagnostic techniques [7].

Drug-resistant bacteria can circulate in populations of humans and animals, through food, water and the environment [6]. The transmission of AMR is facilitated by trade, travel and both human and animal migration. Resistant bacteria can be found in food animals and other food products destined for consumption by humans [8]. Since the need for protein of animal origin is increasing based on the increased human population, the use of antimicrobials in food animals as growth promoters, for infection prevention and treatment is approximately four times higher than the use of antimicrobials in humans [6].

Farmers, particularly broiler producers, utilise many antimicrobials by mixing with feed and/or water with a resultant high prevalence of AMR in broiler meat compared with other animal products [9]. Using antimicrobials in the animal production sector results in AMR and drug residue in the public and animal health sectors [10,11]. Infections caused by antimicrobial-resistant bacteria are becoming more difficult to treat; hence, patients are exposed to longer period of stay in hospital, non-affordable and toxic last-resort drugs and sometimes unsuccessful surgical operations [11].

Aquaculture in Tanzania has a vast yet untapped potential. The industry is dominated by freshwater fish farming in which small-scale farmers practice both extensive and semi-intensive fish farming. In Tanzania, aquaculture was largely regarded as a part-time activity involving about 17,100 fish farmers (14,100 involved in freshwater fish farming and about 3000 in seaweed farming). Aquaculture is one of the fastest growing production sectors in Tanzania, where the current production stands at 11,000 metric tonnes (MT) per year. With lessons learned from Norway, it is feasible to increase productivity in aquaculture through vaccination programs and sound bio-security measures without the use of antimicrobial agents. The activities implemented in Norway that contributed to the drastic reduction in antimicrobial use were (i) the implementation of a unique government industry initiative from the early 1990s to facilitate vaccination against classical furunculosis, (ii) the development of high-quality vaccines by the pharmaceutical industry, (iii) the continuing predominance of vaccination strategies for disease control among fish farmers, (iv) the adoption of ‘all-in-all-out’ production systems with mandatory break periods between year classes, and (v) the zoning and the spatial re-arrangement of marine production sites to minimise the horizontal spread of infections [12,13].

The Codex Alimentarius, which is a collection of internationally adopted food standards and related texts presented in a uniform manner [14], has indicated that AMU governance that minimises the development and transmission of AMR along the food chain is vital, and it encourages the food control authorities to tackle AMR through the introduction of a range of standards related to AMR, veterinary drugs and their residues, food hygiene and animal feed [14]. Similarly, the tripartite collaboration of the World Health Organization (WHO), Food and Agriculture Organization of the United Nations (FAO) and World Organisation for Animal Health (OIE) have set action plans to minimise AMU along the food chain [15,16,17], and these have guided countries to set their own AMU governance legislations. As part of the AMU governance systems, promoting good practices such as proper waste management, animal husbandry and alternative health managements (vaccination and biosecurity) are strongly recommended [15]. As the assessment of the governance of AMU along the food chain is the foundation for the determination of necessary actions for the AMR mitigation, and because previous situation analysis study in Tanzania has indicated that the use of antimicrobials in the broiler sector is high, this sector was purposively selected for this study. The target stakeholders were broiler value chain actors (feed manufacturers, farms and slaughterhouses), input suppliers (veterinary pharmacies) and antimicrobial use regulators (higher and medium level antimicrobial use regulatory institutions). The assessment focused on both the vertical and horizontal interactions of the broiler value chain actors, input suppliers and antimicrobial use regulators.

Crop production accounts for 55% of agricultural GDP and the main export crops are sugar, coffee, cotton, tobacco and tea. While the most common staple crops in Tanzania include maize, cassava, rice, sorghum and millet, the cash crops are maize, cassava, sweet potatoes, bananas, sorghum and sugar cane. Antimicrobials find their way into crops through the following means: insecticide and antifungal spray in crops, animal-sourced manure used to fertilise lands for cropping, antimicrobial-laden waters from sources of effluent pollution (waste dumps, abattoirs and domestic wastewater from septic tanks and pit latrines) used for vegetable farming [18]. It should be noted that Tanzania developed a roadmap for the mitigation of AMR with seven strategic milestones covering the situation analysis up to the implementation of national action plan on AMR, and to date, progress in this regard is monitored closely and reported regularly to the governance structure of AMR in Tanzania, the Multisectoral Coordinating Committee (MCC).

The general objective of this review was to assess the governance of AMU, establish the pattern and extent of AMU, antimicrobial residues (AR) and AMR in food animals and crop agriculture value chains, and relate to strategies in place for combating the emergence of AMR in Tanzania. Specifically, the current study will i) determine the extent of AMR in isolates from food animals and crop agriculture, ii) establish the status of AR in food of animal and plant origins and iii) assess the regulatory oversights related to AMU, AR and AMR in Tanzania.

## 2. Results

A total of 18 establishments were visited between June 2016 and June 2019. A total of 32 field observations/personal interviews, 11 key informant interviews and one group discussion were held involving a total of 83 persons (Table S1).

### 2.1. Farm Practices and the Availability of Antimicrobials in Livestock Farming

Farmers buy antimicrobial (AM) agents to treat animals without the prescription of animal health experts, and AM management is characterised by incorrect dosages, misuse, incorrect applications and non-adherence to withdrawal periods (minimum period of time from administration of the last dose of medication and the production of meat or other animal-derived products for food, when the product should not be consumed by humans or other animals).

Antimicrobial agents are generally used in all domestic animals, and to a limited extent in the wildlife and aquaculture sectors. The most commonly used classes of antimicrobial agents in animals in Tanzania are tetracycline, sulphonamides, penicillin, macrolides and others including antiprotozoal agents (Table 1). During this review, it was reported that animals bought for slaughter from the auction markets were stabilised with antibiotics during trucking to their destination. Animals are injected with oxytetracycline (OTC) in order to prevent transit fever caused by *Pasteurella multocida*. After the application of this drug, withdrawal periods were not observed at all (Table 2).

The primary reasons why farmers use antimicrobials are for sickness prevention (60%), growth promotion (26%) and treatment (14%). Farmers usually use antimicrobials (61%) and they sometimes use other disease prevention techniques such as biosecurity and vaccination (39%) [10,19]. The farmers confirm that the poor biosecurity adoption and implementation in their farm exposes the broiler to diseases. As a result, they repeatedly use antimicrobials to avoid the risk of mortality.

The farmers contact the animal health professionals only if they cannot solve the problem through ‘trial and error’ self-medication and they do not ask for professional advice regularly; instead, they prefer to interact with drug sellers and suppliers directly. If the farmers need to administer antimicrobials, they can purchase them from the nearby veterinary or human pharmacy, drug store or clinic without prescription from the veterinary experts. All members of the farmers group agreed that they individually administer antimicrobials by mixing with feed and water in the dose range of the drug sellers’ recommendation.

The farmers administered antimicrobials when necessary, regardless of the withdrawal period, but antimicrobial such as oxytetracycline (20%) challenges their market, since the color and smell of the broiler meat resembles the drug and is not accepted by the consumer. According to the farmers, the animal health professionals themselves were prescribing antimicrobials for prevention and growth promotion purposes. It was also reported that animals bought for slaughter from the auction markets are stabilised with antibiotics during tracking to their destination.

### 2.2. Antimicrobials in Aquaculture

With the growing trend of aquaculture production in Tanzania, and in the absence of vaccines and formal biosecurity principles and protocols, the use of antimicrobial agents meant for poultry in the treatment of diseases in fish have increasingly been observed. Diseases and infections including broken head syndrome in African sharp tooth catfish, bacterial heamorrhagic septicaemia due to *Aeromonas hydrophila* infection, Pseudomonas infections, *Edwardsiella tarda* infections, as well as *Streptococcus iniae*, *S. agalactiae* and *Lactococcus spp* infections attract the use of antimicrobials. Fertilization of ponds using cattle and poultry manure from treated animals is regarded as an indirect source of antimicrobial agents and their metabolites in aquaculture [20]. No study has established the extent of wash offs, run offs, and leaching from animal and crop farms into aquaculture farms/ponds and, therefore, this is not established in this study due to dearth of information.

In aquaculture, there is a variable extent of resistance of about 70% and the resistance observed was due to environmental contamination. The origin of the pathogens is anthropogenic through human and animal faeces and urine with resistant pathogens or drugs and their residues. Prominent genes encoding resistance to tetracycline, trimethoprim, amoxicillin, streptomycin, chloramphenicol and erythromycin were identified in integrated fish farming systems in the country.

### 2.3. Antimicrobials in Animal Feed

In addition, antimicrobial agents are used in feed formulations; commonly used ones include tetracycline (chlortetracycline and oxytetracycline), colistin and neomycin. Furthermore, additional supplementation with drugs such as neomycin-oxytetracycline preparations for chicks through water is also a common practice in the country. In Tanzania, the Grazing Land and Animal Feed Resources Act No.13 of 2010 [21], give power to the director responsible for grazing lands utilization and animal feed resources, the “Competent Authority” to regulate and control the use of feed additives. In addition, during this study, the formulation of animal feeds by farmers’ themselves or unregistered feed formulators was observed as a common practice. The complexity of the matter is due to the fact that the feed additives, including coccidiostats and antibiotics, when used in feeds, do not require prescription from Veterinarians as stated in the Tanzania Food and Drugs Authority (TFDA; now referred to as the Tanzania Medicines & Medical Devices Authority (TMDA)) guideline for the Registration of Premises for Essential Veterinary drugs of 2015. Since, through feeds, the coccidiostats and antibiotics, though in low concentrations, are used for longer durations, they are considered to be riskier than those used in high doses for disease therapy. Over 90% of drugs and animal feeds sold and used in Zanzibar are imported from mainland Tanzania; hence, effective preventive and control measures in the mainland will also have a positive impact in Zanzibar.

However, regulatory bodies in Tanzania have not yet set standards to regulate the use of growth promoters in the livestock production systems. This is compounded by a lack of One Health monitoring frameworks in the rational use of antimicrobials in the human, livestock, agriculture and environmental sectors.

### 2.4. AMU in Human Health

In Tanzania, HIV/AIDS, malaria, lower respiratory infections (pneumonia) and diarrheal diseases are the top four diseases contributing to illness and death in Tanzania [19,20]. Of these four, bacterial pneumonia and diarrheal infections are the only ones requiring antibiotics for treatment, yet antibiotics are often prescribed for all of them. In addition to the top four diseases, antibiotics are highly used in bloodstream infections including typhoid, sepsis, meningitis and bacteremia, urinary tract infections, sexually transmitted infections (gonorrhea and other bacterial infections), and healthcare-associated infections (such as methicillin-resistant Staphylococcus aureus or MRSA). While many bacterial infections are not part of the general list of the top causes of disease-associated death, their contribution to the growing threat of antibiotic resistance and their effects on overall antibiotics use require further attention among health professionals, patients and consumers, and especially the government. In most cases, similar antibiotics are shared between the public health, animal health and the food sectors [10,22].

## 3. Discussion

### 3.1. Antimicrobial Use (AMU)

In 2010, the global consumption of antimicrobials in the food animal production system was estimated at 63,151 MT and was projected to rise by 67% to 105,596 MT by 2030 [23]. Apart from their utilization for the treatment of animal diseases, worldwide, antimicrobials are also used at sub-therapeutic levels as growth promoters, a practice that contributes significantly to the emergence of AMR.

A study in Morogoro revealed high usage of antimicrobials for therapy and as an animal feed additive, particularly in poultry feeds. The study findings indicated that oxytetracycline, penicillin and streptomycin combination (penstrep), tylosin and sulphonamides were by far the most commonly used antimicrobial agents in cattle in the study area [19]. Furthermore, some of the prohibited antimicrobial agents for animal use, such as furazolidone and chloramphenicol, were found in some veterinary stores and in poultry farms. A significant proportion of Tanzanian poultry farmers treat their chicken by themselves [22,24,25].

In 2009, 90% of broiler farmers interviewed in Morogoro reported high and frequent use of tetracycline, amprolium, sulphonamides, trimethoprim, neomycin and flumequine in their chicken flocks, and 100% of them slaughtered their broilers before the end of the withdrawal period ([19,25], Table 2). Most of the chicken farmers involved in the study in Morogoro used antimicrobial agents for the prevention and treatment of common chicken diseases, namely fowl typhoid, infectious bursa disease (Gumboro), infectious coryza, collibacilosis, coccidiosis, Newcastle disease, helminthosis and fowl pox. Antibiotics accounted for 85% of the drugs used in the farms by volume [19,25]. Another study by Katakweba et al. [26] reported that 40% of Tanzanian small-scale livestock keepers did not know that antimicrobial agents used in animals could pose any risk to human health.

Conventional antimicrobial agents for the prophylactic treatment of bacterial diseases of plants are limited in availability, use and efficacy, and their therapeutic use is largely ineffective. Where studies have been conducted, most applications have been by spray treatment using tetracycline and streptomycin. Use of antimicrobial agents in particular against viral diseases has been recorded in horticultural crops including vegetables [27,28] and cashew nuts. Sulphur dust is widely used for controlling powdery mildew disease caused by *Oidium anacardii* Noack. Increasingly, there is also a high use of low-quality water from wastewater treatment plants in urban areas for horticulture. This is an important route that predisposes consumers to the risk of exposure to antimicrobial agents and the associated residues [29,30,31].

### 3.2. Antimicrobial Resistance (AMR)

The available data from limited studies show a number of multidrug-resistant bacteria that cause mastitis in lactating cows [31,32,33,34]. High levels of resistance have been reported to penicillin G, chloramphenicol, streptomycin and oxytetracycline among *Actinobacter pyogenes*, *Staphlococcus hyicus*, *Staph. intermedius* and *Staph. aureus* from cattle with mastitis. Similar resistance results to amoxicillin + clavulanate, sulphamethoxazole and neomycin have been found in poultry products contaminated with *E. coli* isolates [31,32]. Moreover, there is an increasing trend in the incidence of antimicrobial resistance with a significant increase in multidrug-resistant *E. coli*, *Klebsiella pneumoniae*, *Staph. aureus* and Salmonella in food animals in Tanzania [32,33,34]. The same authors indicated an increase in methicillin-resistant *Staph. aureus* (MRSA) and extended-spectrum beta-lactamase (ESBL) in the food animal sector in Tanzania. Similarly, a high prevalence of antimicrobial-resistant *E. coli* and *Campylobacter spp* isolates from animals have been reported in Tanzania for ampicillin, augmentin, gentamicin co-trimoxazole, tetracycline, amoxicillin, erythromycin, cefuroxime, norfloxacin and ciprofloxacin [32,33].

Antimicrobial resistant Campylobacter in important food animals in Tanzania was reported in isolates from pigs, dairy and beef cattle with specific resistance to ampicillin, gentamicin, streptomycin, erythromycin, tetracycline, ciprofloxacin, nalidixic acid, azithromycin, chloramphenicol and tylosin [35,36,37]. Another study reported the resistance of Campylobacter isolates from ducks in Morogoro to cefuroxime sodium, tetracycline, ampicillin, erythromycin, gentamicin, cloxacillin and amoxicillin [38]. In 2014, other studies revealed a high number of resistant *E. coli* and Enterococci isolates from wildlife and cattle in Tanzanian wildlife ecosystems [39]. Antimicrobial resistance has also been reported in isolates from fish and aquatic environments [40,41].

### 3.3. Antimicrobial Residues (AR)

A study by Jabbar and Grace [42] revealed that antimicrobial residues are present at high levels in foods of animal origin in Tanzania. In support of this observation, Nonga et al. [19] reported antimicrobial residues in 76.4% of 72 broiler meat samples from Zanzibar. In a similar study in the Morogoro municipality, it was found that all 70 egg samples from a commercial poultry farm were positive for antimicrobial residues [43]. In another study on broiler chickens, the same researchers revealed that 70% of sampled farms had antimicrobial residues in their chicken meat [38]. In 2006, Kivaria and colleagues [32] reported antimicrobial residues in raw milk marketed by smallholder dairy producers in Dar es Salaam. These animal-sourced food-associated residues have implications for human health and longer hospitalizations as these organisms may pass through the human food chain to infect humans and lead to drug resistance in humans. Similar microorganisms have been reported in humans [44,45].

Moreover, Kurwijila et al. [33] reported antimicrobial residues in about 36% of milk marketed across Tanzania, suggesting an average risk of about 11 exposures per month for a daily consumer of milk. In addition, Mdegela et al. [34] reported the contamination of milk with antimicrobial residues in smallholder dairy farms in Mvomero and Njombe districts. Using liquid chromatography-mass spectrometry (LC-MS), a high proportion of beef samples in Dodoma had oxytetracycline residues [36,37]. Oxytetracycline residues were reported at 78% in ready-to-eat beef, of which 25.7% had violative residue levels above the maximum residue limits recommended by the FAO and the WHO [14,46,47].

Similar observations were made in studies carried out by Kimera et al. [46] who reported oxytetracycline residues in 71.1% of muscle, liver and kidney samples from slaughtered cattle, of which 68.3% had residues above acceptable regulatory levels. Another study by Bilashoboka et al. [47] demonstrated that 53% of 137 muscle samples from cattle had violative levels of over 0.2 mg/kg. In the same study, 65% of 20 liver samples had levels above tolerable levels of 0.6 mg/kg; and 7.1% of 14 kidney samples had levels above tolerable levels of 1.2 mg/Kg.

### 3.4. Farm Practices and Implications for Antimicrobials Resistance

A number of studies reported that farmers in Tanzania do not observe withdrawal periods as directed by the government policies and drug manufacturers’ recommendations. This is due to ignorance on the associated public health risks and perceived economic losses [25,26,47,48,49,50]. Caudell et al. [23] reported a high use and self-administration of antimicrobials in cattle herds in northern Tanzania. The study also revealed low adherence to withdrawal periods due to poor awareness. These individuals consume meat and milk originating from antimicrobial-treated animals, especially among the Maasai, Arusha and Chagga herders. Similar observations were reported among smallholder poultry farmers in Morogoro where 85% of them were unaware of possible side effects of antimicrobial residues in humans [48]. The risks involved in the consumption of contaminated food of animal origin with antimicrobial residues (AR) was found to be due to the consumption of animal products directly from the farm as well as due to non-adherence to withdrawal periods.

Farmers fail to observe and adhere to withdrawal periods due to perceived economic losses. For instance, a withdrawal period of 28 days for tylosin (20%) and 45 days for gentamycin seems impractical (Table 2) and findings by Kimera et al. [46] and Mgonja et al. [37] support this observation. Similarly, the withdrawal periods for different drugs for eggs and broiler meat seem rather difficult to observe from an economic viewpoint, an observation that is supported by findings by Nonga et al. [19,25]. As mentioned earlier, the withdrawal period is the minimum period of time from the administration of the last dose of medication and the production of meat or other animal-derived products for food, when the product should not be consumed by humans or other animals. In this case, such consumption of meat, milk and eggs mentioned above leads to the transfer of antimicrobial residues to humans through the food chain.

During this study, it was also reported that animals bought for slaughter from auction markets are stabilised with antibiotics during trucking to their destination, particularly with oxytetracycline (OTC), and this might be the reason that OTC has been detected in beef in different studies [36,37,46,47].

### 3.5. Diagnostics, Governance and Supplies of Antimicrobials

The Tanzania Veterinary Laboratory Agency (TVLA), established in 2012, is mandated to undertake the diagnosis of animal diseases, regulate veterinary laboratories, conduct research on animal diseases and vectors, and develop and produce vaccines and other biologicals. The agency offers laboratory diagnostics services throughout the country through its eight diagnostic centers. In addition to TVLA, diagnostic services for animal health are also provided by Sokoine University of Agriculture (SUA) through its teaching and research laboratories. During this study, it was found that most of microbiology laboratories are poorly supplied with equipment and reagents for microbial identification and testing for resistance.

The supply chain of veterinary medicines from the sources to the end users is carried out by the private sector. The government is mainly involved in regulatory and monitoring functions through the Tanzania Medicines and Medical Devices Authority (TMDA) and the Veterinary Council of Tanzania (VCT). The supply chain of veterinary medicines include the source (manufacturers and importers), distributors and end users. Antimicrobial agents are imported from Europe, mainly Belgium, the Netherlands, France and Turkey, as well as Asian countries such as China, India and Indonesia. Between 2009 and 2017, the major companies importing veterinary medicines were Twiga, Chemical Industries, Bytrade (T) co. Ltd., Norbrook, Farmers Centre, Farm Base, Anicrop Services, Tan Veterina, Ultravetis, Cooper Tanzania (TFDA, 2017) and Bajuta. Once imported, they are distributed to sellers and eventually to the end users of antimicrobial agents.

**Figure 1:**
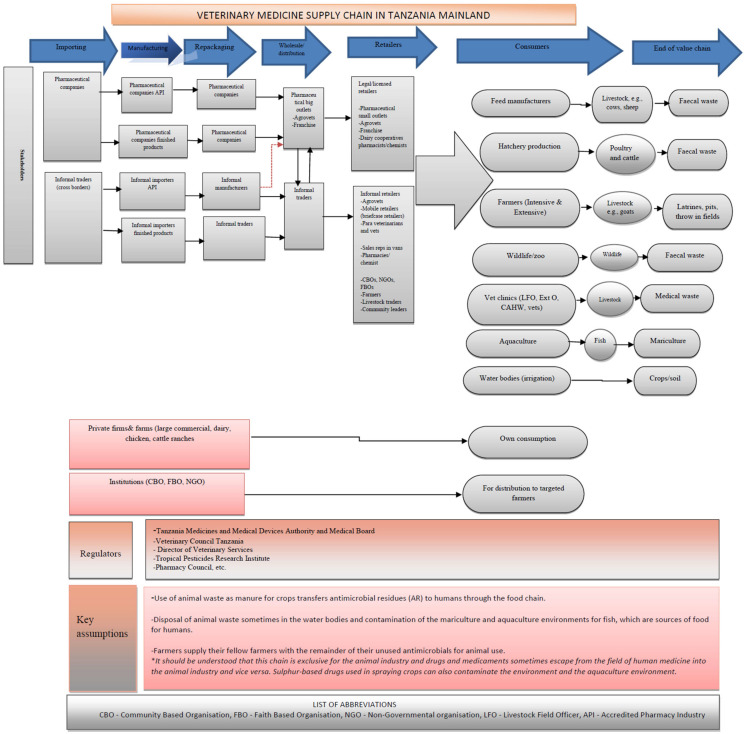
The supply chain covering different nodes in the pathways from manufactures to the clients as end users of Veterinary pharmaceuticals in Tanzania.

As demonstrated in Figure 1, the client can receive the antimicrobial agents through legal and informal pathways. Access to antimicrobial agents through informal pathways contributes significantly to the misuse and associated residues in food of animal origin and the perpetuation of the development and spread of antimicrobial resistance to animals, and consequently, to humans [10,21,38].

While the quality assessments are performed to a great extent on imported veterinary drugs at the port of entry, there is very weak post-market surveillance. The supply chain is driven by the private sector, and thus, it is not uncommon to find importers, distributors and wholesalers supplying drugs directly to consumers, a route that is considered informal [10,32,51]. With weak regulations, antimicrobial agents are commonly sold during auction markets for livestock by informal vendors such as petty traders and livestock keepers. Antimicrobial agents found in markets such as these are often unregistered and are therefore sold at cheap prices. The quality of these medicines is also difficult to determine because access to them is difficult.

The delivery of animal health services and the dispensing of veterinary medicines to consumers is governed by the Veterinary Act No. 16 of 2003 [52]. In Tanzania, the Animal Health work-force responsible for the delivery of animal health services includes 700 degree-holding veterinarians, over 1500 veterinary paraprofessionals (diploma level) enrolled and 1340 veterinary paraprofessional assistants who hold certificates and are enlisted by the Veterinary Council of Tanzania (VCT). Another 60% of veterinary paraprofessionals operate without being enrolled or enlisted. The public veterinary services also includes the Zoosanitary Inspectorate Services (ZIS) established in 1996, which manages 36 border posts, 19 quarantine stations and 381 internal checkpoints. The regulation of the delivery of veterinary services is provided for in the Animal Diseases Act, 2003 and Veterinary Act of 2003 [52,53].

Tanzania is endowed with different capacities for researching and monitoring the state and trends of antimicrobial resistance. Such capacities, however, are based primarily in research and training institutions. The list of institutions with these capacities and that are conducting research on AMR with a focus on humans, animals and the environment are as listed in Table 3.

The animal health care system in Tanzania is organised from the primary level (at the farm or community level), secondary level (district level) and tertiary level (with services provided by Sokoine University of Agriculture, the only University undertaking veterinary training in the country). Following the privatization and decentralization of veterinary services in the 1990s, documentation and record keeping was rather disorganised. Thus, the information on AMU and testing for resistance and residues is non-existent.

The National Sample Survey indicates that only 20% of Tanzanian farmers utilise extension services [1], and given the malpractice observed, it is prudent that farmers’ awareness of AMU and AMR is ensured, requiring a reliable extension service system.

Some developed countries have banned the use of antimicrobial agents as feed additives to prevent and control the emergence of AMR since the 1980s. In Sweden, for instance, legislations were enacted in 1986 banning the use of antimicrobials as growth promoters (AGPs); thus, the use of veterinary medicines in animals became restricted to prescription only by veterinarians. Moreover, in the European Union, a total ban on the use of antibiotics as growth promoters was introduced in 2006. Such interventions, however, are lacking in developing countries including Tanzania.

The Veterinary Act No. 16 of 2003 [52], provides for the regulation of veterinarians and paraprofessionals; however, the law also provides circumstances under which unqualified personnel may perform some of the duties. During the study, it was observed that the exceptions provided are misused by unqualified personnel to deliver veterinary services such as disease diagnosis and treatment including self-medication administered to animals by owners and other farm attendants.

The withdrawal period is defined as the interval between the last administration of a drug to the animal under normal condition of use and production of foodstuff from such animals. This is a requirement in food-producing animals to ensure that such foodstuffs do not contain residues in quantities exceeding the maximum accepted residue limits. Adherence to withdrawal periods is perceived negatively by farmers due to high economic losses from discarded milk during and after the treatment of diseases such as mastitis in high-producing animals. In poultry production, for instance, there is a high prevalence of fowl typhoid and other bacterial diseases, as well as coccidiosis that could have been minimised if practical bio-security measures instead of antimicrobial agents were available during the entire production cycle. In addition, an inadequate number of veterinary service providers and the inadequate regulation of service provisions aggravate the challenge.

The application of animal manure, which is regarded as a good source of nutrients, is a common practice in crop agriculture, as well as in aquaculture. Animal manure from intensive production systems often contain antimicrobial agents resulting from extensive therapeutic and sub-therapeutic uses. Since the absorption of antimicrobial agents in the animal gut is never complete and since substantial amounts are excreted through urine and faeces that end up in manure, this exposure scenario should be considered in the risk analysis.

Though scarcely studied in Tanzania, there are potential human health risks associated with the consumption of fresh vegetables, water and fish contaminated with manure with antimicrobial agents. Such risks may be higher for consumers who are allergic to antimicrobial agents. The same pathway may also contribute to and enhance the development of antimicrobial resistance. In Tanzania, both hospital- and community-acquired bacterial infections have been widely reported across the country [21,40,54], especially from *E. coli*, *Klebsiella spp, P. aeruginosa* and *Staph. Aureus*, which are predominant in urinary tract infections (UTI), blood stream infections (BSI) and skin and soft tissue infections (SSTI) [21,40]. Furthermore, *Salmonella spp* and *Campylobacter spp* have been predominantly isolated in food- and water-borne gastrointestinal infections, as well as Vibrio cholera in cholera outbreaks and Mycobacterium tuberculosis in chronic respiratory tract infections [21,44,45]. These isolates, causing UTI, BSI and SSTI, were resistant to widely available antimicrobials such as ampicillin, trimethoprim-sulphamethozaxole and chloramphenicol [21,54]. In addition, the increasing burden of the antimicrobial resistance to penicillins, cephalosporins and clavulanate in human therapy has been reported [54,55,56]. Similarly, food-borne and invasive salmonellosis as well as typhoidal salmonellosis and campylobacteriosis have been documented in Tanzania with resistance to chloramphenicol, ampicillin and cefuroxime, among others [57]. It should be noted that most of these medications mentioned above are shared in the human and animal health sectors and the inter-species vertical or horizontal transfer of resistance may have occurred.

The national strategy to reduce the threat of AMR in food animals and crop agriculture should consider the prudent use of antimicrobial agents, improvement of the capacity for disease diagnosis and the detection of antimicrobial residues, development of bio-security guidelines that fit into current production systems, and control of antimicrobial residues. The National Action Plan on Antimicrobial Resistance 2017 to 2022 [58] outlines key strategic objectives, interventions and activities to slow down the development and spread of AMR and improve patient outcomes.

## 4. Materials and Methods

### 4.1. Study Design, Sampling Strategy, Study Coverage and Data Collection

**Figure 2:**
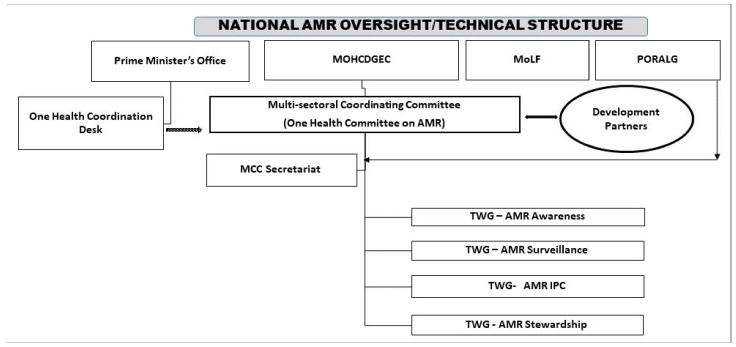

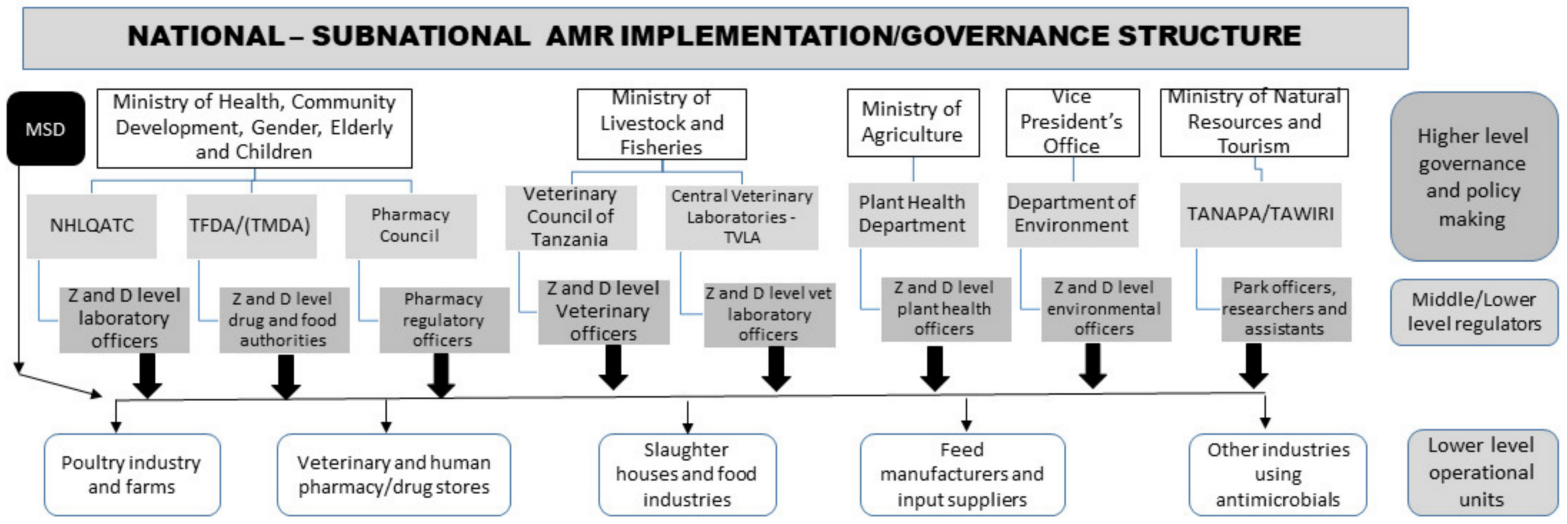
The antimicrobial-related governance structure for the food and agriculture sector in Tanzania. MoHCDGED = Ministry of Health, Community Development, Gender, Elderly and Children; MoLF = Ministry of Livestock and Fisheries; PORALG = President’s Office Regional Administration and Local Government; TWG = Technical Working Group; MCC = Multi-sectoral Coordinating Committee; IPC = Infection Prevention and Control; MSD = Medical Stores Department; NHLQATC = National Health Laboratory Quality Assurance and Training Centre; TFDA/TMDA = Tanzania Food and Drugs Authority, now called Tanzania Medicine and Medical Devices Authority; TVLA = Tanzania Veterinary Laboratory Agency; TANAPA = Tanzania National Parks Authority; TAWIRI = Tanzania Wildlife Research Institute; Z&D = Zonal and District (these are the secondary and tertiary level units of government in the United Republic of Tanzania).

A multimethod approach was used, including a desk review, focus group discussions (FGDs), key informant interviews (KIIs) and field observations. Identification of all stakeholders in the governance structure was conducted through a rapid survey of the current documents and physical visits were made to relevant establishments located in the Dodoma, Arusha, Morogoro and Dar es Salaam regions (Figure 2). During the visits, a range of information was collected in order to map out the state, trends and magnitude of antimicrobial use in food animals and crop agriculture, antimicrobial resistance as well as antimicrobial residues. The analysis and interpretation of the data collected was referenced to FAO and OIE strategic objectives for combating AMR [15,16]. The establishments/institutions visited were purposely selected due to their relevance to the subject matter. This review of the food and agriculture sector was meant to complement previous situational analyses and research conducted on AMR, AR and AMU in Tanzania, particularly in the human health sector [21,27,32,40,49]. The list of grey literature was obtained from the legal documents archives and the legislation that is directly or indirectly related to AMU and AMR (Table S2). A total of eight documents from the grey literature were reviewed and analyzed. The national and sub-national implementation and governance structures have been elaborated in Figure 2 below.

It should be noted that while this structure under consideration relates primarily to the food and agriculture industries, the broader health industry has a public health system referral pyramid, which also influences antimicrobial use and antimicrobial resistance in humans [44,45]. Details of other associated regulatory bodies that influence issues related to AMR in Tanzania are available (see Supplementary Materials; [45]).

Secondly, a qualitative cross-sectional field study on the situation of AMU governance along the broiler value chain was conducted from April to June 2019. The components of this study were FGDs with broiler farmers and KIIs with high- and medium-level AMU regulatory experts and further grey literature reviews of AMU and AMR legislations in Tanzania. The regulatory bodies of AMU were directly chosen for the key informant interviews. The responsible experts from each regulatory institution were identified and invited for interview with the help of the Emergency Center for Transboundary Animal Diseases (ECTAD) Tanzania. To double check the information, field evaluations of the broiler value chain were conducted to complement the FGDs and KIIs. During the field observations, informal (non-structured) interviews were conducted based on the AMU phenomena and driving factors for AMR. By using these methods, the knowledge, perception and practice of the broiler value chain actors, input supplier and antimicrobial use regulators about AMU and AMR were assessed and analyzed for AMU governance.

Using the updated AMU situational analysis in the food and agriculture sector, [10], study areas were chosen based on the following criteria: (i) a high human population density, (ii) a high number of broiler farms, (iii) the existence of regulatory bodies and (iv) the presence of broiler farm input suppliers. Specifically, cities in Dar es Salaam, Dodoma, Morogoro and Arusha were purposely selected as the study areas. The study participants for the field study were chosen by a snowball sampling method, a situation in which one food chain actor (broiler farmer, feed producer, slaughterhouse worker or veterinary pharmacist) interviewed referred the researchers to another actor. In this way, the data collection was continued until the information saturation point was reached. For the FGDs at the broiler farms, participants were chosen randomly from the list provided by the broiler farmers association using the inclusion criteria ‘minimum of secondary level education’ to avoid peer dominancy during the discussion and to ease communication. In total, eight broiler farms, 11 veterinary pharmacies, seven slaughterhouses (three abattoirs and four open areas) and six broiler feed manufacturers were visited to conduct one focus group discussion and 11 KIIs.

The situational analysis on the AMU, AR and AMR in food animals including fish farming (aquaculture) and crop production was carried out in 19 institutions in mainland Tanzania. The regions and locations visited for data collection were Arusha, Dar es Salaam, Dodoma and Morogoro together with Unguja in Zanzibar.

### 4.2. Focus Group Discussions and Key Informant Interviews

The FGDs, which were conducted with a group of broiler farmers, lasted for three hours. This was designed to extract information using a relational diagram, impact analysis and trend analysis. By using these methods, participants were facilitated to model, quantify, estimate, compare and rank categories. Proportional piling was used to prioritise categories and parameterise. The respondents prioritised and ranked the purpose of AMU and disease prevention techniques on their farms. During ranking, the farmers were not in agreeance, and they complained as they had different AMU practices. Farmers prioritised individually and the mean values were calculated for all quantities.

The regulatory bodies that directly or indirectly control the use of antimicrobials in the broiler value chain were chosen for the key informant interviews. For these interviews, checklists were used as a guiding tool for the interviewers. The specific organizations included (i) the Prime Minister’s office—One Health coordination desk; (ii) the Central Veterinary Reference Laboratory for Tanzania; (iii) the Ministry of Livestock and Fisheries; (iv) the Tanzanian Veterinary Laboratory Agency; (v) the Ministry of Health, Community Development, Gender, Elderly and Children; (vi) the Ministry of Agriculture; (vii) the Veterinary Council of Tanzania; (viii) both the zonal and national Tanzanian Food and Drug Authority (now named the Tanzania Medicine and Medical Devices Authority); and (x) the Dodoma Municipality Veterinary Officer. From each stakeholder, information about the available and planned legislations of AMU, activities for the governance of AMU and its challenge, knowledge and perceptions of AMU externalities and their recommendation about the mitigation of AMU were collected.

### 4.3. Field Observations

**Figure 3:**
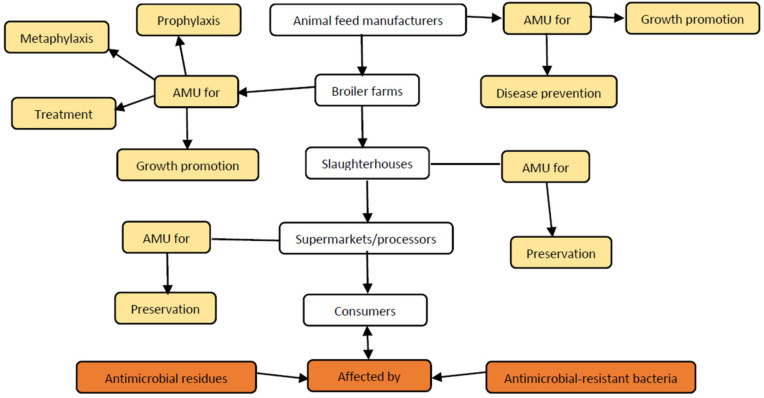
Potential antimicrobial use, development of AMR/residue and transmission model.

Observation is the selection and recording of users’ behavior in the working environment. The field observations were conducted in the broiler business operations including feed producing companies, broiler farms, broiler slaughterhouses (including open slaughter areas) and veterinary pharmacies. Field observation of the broiler food chain was conducted starting from animal feed to the final end product of broiler meat. For instance, the hygiene of the production environment, the input used for production (addition of antimicrobial), manure management, the reason for AMU in each stage of the food chain, the application procedure of antimicrobials and availability of alternatives (biosecurity and vaccination) were among the focus issues of the informal interviews. By using the five sense organs, valuable and valid information was collected and associated with the governance of AMU in that particular context. Before the fieldwork, the expected AMU and AMR creation and transmission model was explained, as shown in Figure 3.

### 4.4. Grey Literatures That Provide Legislative Tools Guiding Antimicrobial Use in Animals in Tanzania

Legislation such as the (i) Tanzania Foods, Drugs and Cosmetics Act of 2003, Part IV (2003); (ii) Tanzania Pharmacy Act (2011); (iii) Tanzania Animal Diseases Act 17 of 2003, Section 50 (2003); (iv) Tanzania Grazing Land and Animal Feed Resources Act (2010); (v) Tanzania Veterinary Act (2003); (vi) Tanzania Water Supply and Sanitation Act (2009); (vii) Tanzania Animal Welfare Act (2008); and (viii) Tanzania Public Health Act (2009) were used as sources of information (Supplementary Table S2). Other legislative frameworks include the (i) Fisheries Regulations of 2009, Section 33 (f); (ii) Fisheries Regulations of 2009, Section 33 (i); (iii) Fisheries Regulations of 2009; Section 33 (j–l); and (iv) the harmonised EAC SPS measures (under review) Section 2.8-(2.8.1), Section 3.7-(3.7.5), Section 3.7-(3.7.6) and Section 3.12 [37,38,47,48,49]. The focus of this grey literature review was to gather information related to the available AMU and/or AMR legislation in each level of the food chain and to cross-check the implementations of these legislations in the lower broiler business operations.

### 4.5. Data Analysis

Qualitative analysis was predominantly used and some data are expressed in numbers. Those numerical values were expressed in descriptive statistics (figures, tables and percentages). The data collected from the group discussions, field visits, key informant interviews and grey literatures were interpreted in terms of antimicrobial use governance in the food chain. The information recorded in the forms of texts, photos and audio were textually and semi-quantitatively analyzed towards AMU governance. For the textual analysis, a qualitative content analysis approach was used. The contents of grey literatures, recorded audio and visual pictures were analyzed in terms of AMU governance. The contents of grey literatures were categorised as direct and/or indirect based on the concept of their contribution to the governance of AMU and AMR along the food chain.

Some data, such as the result of proportional piling, were collected in numerical form from the field and the quantity was directly taken for analysis. The frequency of repetition of the information from different participants was counted and analyzed quantitatively. The practices of the visited farms were judged using 12 criteria of good AMU, each being classified along three categories (bad, moderate and good). The mean, minimum and maximum scores of these categories were calculated from the practice of the visited farms. The AMU nodes (introduction routes of antimicrobials) were ranked based on the AMU status (usage or absence of antimicrobials) of broiler business operators (broiler feed manufacturers, broiler farms and broiler slaughterhouses).

## 5. Conclusions

From this study, it can be concluded that the effective control of AMR in the food and agriculture sectors in Tanzania requires a thorough understanding of the governance structure on the issue of antimicrobials and concerted efforts of all stakeholders using the One Health approach—a collaborative, multisectoral and transdisciplinary approach—working at local, regional, national and global levels to achieve optimal health and well-being outcomes recognizing the interconnections between people, animals, plants and their shared environments. The delivery of plant/animal health services and dispensing of antimicrobial agents must be streamlined, regulated and carried out by professionals. Service delivery by unqualified personnel should be avoided. Overall, the practical recommendations based on the findings from the study include:

(a) Review of legislation governing AMU, AMR and AR in food and agriculture production systems;
(b) Awareness creation and education interventions to improve the understanding of AMU, AMR and AR in food and agriculture and their subsequent effects to humans and the environment;
(c) Establishment and strengthening of the national surveillance and monitoring programs for AMU, AMR and AR in food and agriculture to enhance burden estimation and for the early detection of emerging resistant pathogens as well as the assessment of effectiveness of control measures;
(d) Development and promotion of biosecurity principles (including good husbandry practices) for food animal production systems;
(e) Promotion of stewardship and legislations on AMU in food and agriculture sectors;
(f) Enhancement of the development and use of rapid and innovative diagnostic tests for “point-of-need” of antimicrobials as well as AR in food products; and
(g) Empowerment of consumers with knowledge, so as to serve as a pressure group and game changer by demanding high-quality products that are free from AR and antimicrobial-resistant pathogens.

## Figures and Tables

**Table 1:** Imported and registered antimicrobials for use in animal health and crop production in Tanzania from 2015 to 2017.

Class of Antimicrobial	Type of Antibiotic/Common Name
Tetracyclines	Oxytetracycline
	Doxycycline + Colistine
	Oxytetracycline + Vitamins
	Chlortetracycline
	Tylosin + Doxycycline
	Oxytetracycline + Colistine
	Oxytetracycline Hydrochloride
Penicillins	Cloxacillin
	Ampicillin + Cloxacillin
	Procaine Benzyl Penicillin
	Penicillin G + Neomycin
	Penicillin G + Dihydrostreptomycin
	Amoxicillin trihydrate
Sulfonamides and antiinfectives	Diaveridine + Sulfadimidine
	Sulfamethaxazole + Trimethoprime
	Trimethoprim + Sulphadiazine
	Sulfamethazine + Trimethoprime
Macrolides	Tylosin
Quinolones	Norfloxacin
	Enrofloxacin
Antiprotozoal	Amprolium HCl + Sulfaquinoxaline + Vitamin K3
	Diminazine Diaceturate IP + Cyanocobalamine BP + Antipyrine
	Diminazine Diaceturate IP + Antipyrine
	Isomedium
	Homidium chloride
	Parvaquone
	Diminazine + Phenazone
	Imidocarb
	Amprolium HCl
	Buparvaquone
	Diminazine
	Diclazuril and Toltrazuril

**Table 2:** The Withdrawal period (in days) for some popularly accessible antimicrobial agents found in the market in the Morogoro municipality, Tanzania, July 2017.

Antimicrobial Agent	Withdrawal Period in Days Indicated in the Labels and Leaflets						
	Beef	Liver /Kidney	Milk	Broiler	Eggs	Pork	Cattle, Sheep & Goats
Penistrep	10						
Tylosin (20%)	28		3				
Gentamycin	7	45	2				
Sulphadimidime + Diaveridine				12	12		
Trimethoprim + Sulphadiazine				12	Do not administer		
Oxytetracycline (50%)				7	0		
Sulfachloropyramine				7	3		
Doxycycline + Colistine				7		8	14
Oxytetracycline (20%) injectable	8			6			
Amprolium Hydrochloride 20% with Vitamin K3				14			14
Chlotetracycline (20%)				7	9		10
Doxycycline + Tylosin				7		8	14
Sulphadimidine sodium 400 mg + Sulphaquinoxaline sodium 150 mg				7		8	14
Doxycycline					21	6	10
Flumesol Flumequine				3		8	
Trimethoprime + Sulphadiazine				12	10		
Sulphaclozime (30%)							
Oxytetracycline + Neomycin + Chloramphenicol				12			
Amoxcillin + Colistine				7			
Doxycline + Gentamycin					14		
Amprolium				3	3		
Triple Sulpha		7		10		14	
Oxytetracycline Hydrochloride 20%					7		
Doxycycline hydrate + Colistine				7			
Ammonium chloride + Magnesium sulphate + Sodium sulphate + Sorbitol				Nil			
Tylosin Tartrate 20%				7			
sulphamezathine (sulphadimethylpyrimidine) + sulphadiazine (sulphapyrimidine)				14			
Oxytetracycline 40%				6		8	8
Trimethoprim + Sulfadiazine				12			
Toltrazuril				12			9

**Table 3:** List of training and research institutions currently involved in AMR research activities in Tanzania.

Institution	Culture and Sensitivity Tests & Techniques	DNA Based Test & Techniques	Animal Focus	Human Focus	Environmental Focus
Sokoine University of Agriculture	+	+	+	+	+
Nelson Mandela African Institute of Science and Technology	+	+	+	+	+
Tanzania Veterinary Laboratory Agency	+	-	+	-	-
Muhimbili University of Health and Allied Sciences	+	-	-	+	-
Bugando Medical Centre	+	+	+	+	+
Kilimanjaro Christian Medical Centre	+	+	-	+	-
National Institute for Medical Research	+	+	-	+	-

## Data Availability

Data utilised in this study are publicly available and are accessible on request.

## Supplementary Materials

The following are available online at https://www.mdpi.com/article/10.3390/antibiotics10040454/s1, Table S1. Regions and locations visited during data collection; Table S2. List of relevant documents, grey literature and instruments related to AMR in Tanzania consulted and references. References [59,60,61,62,63,64,65,66,67,68,69,70,71,72,73,74,75,76,77,78,79,80,81,82,83,84,85,86] are referred to in the Supplementary Materials.

## Abbreviations

AMR	antimicrobial resistance
AMU	antimicrobial use
AR	antimicrobial residue
FGD	focus group discussion
GARP-TWG	Global Antibiotic Resistance Partnership—Tanzania Technical Working Group
GDP	gross domestic product
KII	key informant interview
MoLF	Ministry of Livestock and Fisheries
TLMI	Tanzania Livestock Modernization Initiative
TLMP	Tanzania Livestock Master Plan
TFDA	Tanzania Food and Drugs Authority, now referred to as the Tanzania Medicines and Medical Devices Authority (TMDA)
VCT	Veterinary Council of Tanzania
ZIS	Zoosanitary Inspectorate Services
