# Supplementary material for: Antimicrobial Use, Residues, Resistance and Governance in the Food and Agriculture Sectors, Tanzania
Source: Antibiotics (Basel). 2021 Apr 16;10(4):454. doi: 10.3390/antibiotics10040454 (PMC8073917; doi:10.3390/antibiotics10040454)
Supplement: Supplementary file 1 [file antibiotics-10-00454-s001.zip › antibiotics-1068575-supplementary.pdf]

antibiotics

MDPI

Table S1. Regions and locations visited during data collection.

Location	Establishments visited during the mission
Dar es Salaam	Tanzania Food and Drug Authority
	Plant Protection Department (Division)
Dodoma	Ministry of Livestock and Fisheries Development (MLFD) Directorate of Veterinary Services
	Tanzania Food and Drug Authority (Central Zone)
	Tanzania Veterinary Laboratory Agency (TVLA) Central Zone
	Ministry of Agriculture, Plant Protection Department (Division) Central Zone
	Dodoma Urban Water and Sanitation Authority (DUWASA)
Arusha	Nelson Mandela African Institutions of Science and Technology (NM-AIST)
	East Africa Community (Productive Sectors), Department of Agriculture and Food security
	Arusha Zonal Veterinary Investigation Center (ZVIC)
	Tanzania Veterinary Laboratory Agency (TVLA) Northern Zone
	Meru District Council: District Veterinary Officer (DVO) and District Fisheries Officer (DFO)
	Tropical Pesticide Research Institute (TPRI)
	Aquatic and Veterinary Shop in Arusha
Morogoro	Sokoine University of Agriculture (SUA)
Zanzibar (Unguja)	Directorate of Veterinary Services
	Division of Plant Protection and Produce Inspection
	Zanzibar Food and Drug Agency (ZFDA)

Table S2. List of relevant documents, grey literature and instruments related to AMR in Tanzania consulted and references.

	Reference
Fisheries Act No 22 of 2003.	[1]
Fisheries Regulation of 2009.	[2]
Tanzania Food and Drug Authority (TFDA) Act No 1 of 2003.	[3]
Tanzania Food and Drug Authority (TFDA) Regulation of 2006.	[4]
Tropical Pesticide Research Institute Act No 18 of 1979.	[5]
The Veterinary Act No 16 of 2003.	[6]
The Animal Disease Act No 17 of 2003.	[7]
The Dairy Industry Act No 8 of 2004 Cap 262.	[8]
Animal Disease Regulations of 2007.	[9]

National Livestock Policy, 2006.	[10]
Livestock Sector Development Strategy, 2010.	[11]
Livestock Sector Development Programme, 2011.	[12]
Tanzania Livestock Modernization Initiatives, 2015.	[13]
Environmental Management Act 20 of 2004.	[14]
The Grazing Land and Animal Feed Resources Act No.13 of 2010	[15]
The Grazing Land and Animal Resources Regulations of 2010.	[16]
The Meat Industry Act No10 of 2006.	[17]
Animal Welfare Act No. 19 of 2010.	[18]
The Hides and Skin Trade Act No 18 of 2008.	[19]
National Guideline for Supervision of Livestock Services of 2016.	[20]
The Livestock Identification, Registration and Traceability Act No 12 of 2010.	[21]
The East Africa Community (EAC) - Sanitary and Phytosanitary Protocol.	[22]
Livestock Extension Service Guidelines 2006.	[23]
World Trade Organization (WTO): Sanitary and Phytosanitary Measures Agreements.	[24]
National Action Plan for Antimicrobial Resistance 2017.	[25]
The WHO Global Action Plan for Antimicrobial Resistance 2015.	[26]
OIE Performance of Veterinary Services (PVS) Follow-up Mission Report 2016	[27]
The FAO Action Plan on Antimicrobial Resistance 2016.	[28]
Zanzibar Livestock Policy, 2011.	[29]
Mwongozo wa Usajili wa Maduka ya Dawa Muhimu za Mifugo, 2015. (in Swahili)	[30]

Additional resources [31–33].

References

1. Government of Tanzania. *Fisheries Act, 2003;* Act No. 22 of 2003, Government Press, Dar es Salaam, Tanzania, 42p, Available online: http://extwprlegs1.fao.org/docs/pdf/tan53024.pdf (accessed on 23 February 2021).
2. Government of Tanzania. *Tanzania Fisheries Regulations of 2009;* Act No.2 of 2009, Government Press, Dar es Salaam, Tanzania, 520p, Available online: http://extwprlegs1.fao.org/docs/pdf/vic121353.pdf (accessed on 23 February 2021).
3. Government of Tanzania. *Tanzania Foods, Drugs and Cosmetics Act of 2003, Part IV, 2003;* Act No.1 of 2003, Government Press, Dar es Salaam, Tanzania, 89p, Available online: https://tanzania.eregulations.org/media/TFDA%20ACT.pdf (accessed on 23 February 2021)
4. Government of Tanzania. *Tanzania Food and Drug Authority (TFDA) Regulation of 2006.;* Act No.116 of 2006, Government Press, Dar es Salaam, Tanzania, 11p, Available online: http://extwprlegs1.fao.org/docs/pdf/tan153765.pdf (accessed on 23 February 2021)
5. Government of Tanzania. *Tropical Pesticides Research Institute Act, 1979 (No. 18 of 1979);* Act No.18 of 1979, Government Press, Dar es Salaam, Tanzania, 30p, Available online: http://extwprlegs1.fao.org/docs/pdf/tan4081.pdf (accessed on 23 February 2021)
6. Government of Tanzania. *The Veterinary Act, 2003;* Act No.16 of 2003, Government Press, Dar es Salaam, Tanzania, 31p, Available online: https://www.mifugouvuvi.go.tz/uploads/publications/sw1595836341-The%20Veterinary%20Act,%202003.pdf (accessed on 23 February 2021)
7. Government of Tanzania. *Animal Disease Act;* Act No.17 of 2003, Government Press, Dar es Salaam, Tanzania, 10p, Available online: http://extwprlegs1.fao.org/docs/pdf/tan169399.pdf (accessed on 23 February 2021)
8. Government of Tanzania. *The Dairy Industry Act No 8 of 2004 Cap 262.*, Act No. 8 of 2004, Government Press, Dar es Salaam, Tanzania, 22p, Available online: http://extwprlegs1.fao.org/docs/pdf/tan61559.pdf (accessed on 23 February 2021)

9. Government of Tanzania. *Animal Disease Regulations of 2001, Act No. 131 of 2001*, Government Press, Dar es Salaam, Tanzania, 4p, Available online: http://extwprlegs1.fao.org/docs/pdf/tan28124.pdf (accessed on 23 February 2021)
10. Government of Tanzania. *National Livestock Policy, 2006*, Government Press, Dar es Salaam, Tanzania, 55p, Available online: https://www.tnrf.org/files/E-INFO_National_Livetock_Policy_Final_as_per_Cabinet_Dec-2006.pdf (accessed on 23 February 2021)
11. Government of Tanzania. *Livestock Sector Development Strategy, 2010.*, Government Press, Dar es Salaam, Tanzania, 90p, Available online: https://www.tanzania.go.tz/egov_uploads/documents/development_strategy-_Livestock_sw.pdf (accessed on 23 February 2021)
12. Government of Tanzania. *Livestock Sector Development Strategy, 2011*, Government Press, Dar es Salaam, Tanzania, 123p, Available online: https://www.tanzania.go.tz/egov_uploads/documents/Livestock_Programe_sw.pdf (accessed on 23 February 2021)
13. Government of Tanzania. *Tanzania Livestock Modernization Initiatives, 2015.*, Government Press, Dar es Salaam, Tanzania, 40p, Available online: https://livestocklivelihoodsandhealth.org/wp-content/uploads/2015/07/Tanzania_Livestock_Modernization_Initiative_July_2015.pdf (accessed on 23 February 2021)
14. Government of Tanzania. *Environmental Management Act, 2004, Act No. 20 of 2004*, Government Press, Dar es Salaam, Tanzania, 129p, Available online: http://extwprlegs1.fao.org/docs/pdf/tan61491.pdf (accessed on 23 February 2021)
15. Government of Tanzania. *Tanzania Grazing Land and Animal Feed Resources Act, 2010*; Act No.13 of 2010, Government Press, Dar es Salaam, Tanzania, 32p, Available online: http://extwprlegs1.fao.org/docs/pdf/tan97356.pdf (accessed on 23 February 2021)
16. Government of Tanzania. *The Grazing Land and Animal Resources Regula-tions of 2010;* Act No.13 of 2010, Government Press, Dar es Salaam, Tanzania, 32p, Available online: https://www.mifugouvuvi.go.tz/uploads/publications/en1595835588-GRAZING%20LAND%20AND%20ANIMAL%20FEED%20RESOURCE%20ACT%202010.pdf (accessed on 23 February 2021)
17. Government of Tanzania. *The Meat Industry Act No10 of 2006;* Act No.10 of 2006, Government Press, Dar es Salaam, Tanzania, 19p, Available online: https://trade.business.go.tz/media/The%20Meat%20industry%20Act,%202006.pdf (accessed on 23 February 2021)
18. Government of Tanzania. *Tanzania Animal Welfare Act, 2008;* Act No.19 of 2009, Government Press, Dar es Salaam, Tanzania, 25p, Available online: http://extwprlegs1.fao.org/docs/pdf/tan85327.pdf (accessed on 23 February 2021)
19. Government of Tanzania. *The Hides and Skin Trade Act No 18 of 2008;* Act No.18 of 2008, Government Press, Dar es Salaam, Tanzania, 23p, Available online: https://www.tanzania.go.tz/egov_uploads/documents/The_Hides,_Skins_and_Leather_Trade_Act,_2008_-_(Act_No_sw.pdf (accessed on 23 February 2021)
20. Government of Tanzania. *National Guideline for Supervision of Livestock Services of 2016*, Government Press, Dar es Salaam, Tanzania, 57p, Internal document.
21. Government of Tanzania. *Livestock Identification, Registration and Traceability Act, 2010;* Act No. 12 of 2010, Government Press, Dar es Salaam, Tanzania, 18p, Available online: http://extwprlegs1.fao.org/docs/pdf/tan97294.pdf (accessed on 23 February 2021)
22. East African Community. *EAC Protocol on Sanitary and Phytosanitary (SPS) Measures, 2013;* July 2013, EAC Press, Arusha, Tanzania, 11p, Available online: https://www.eac.int/documents/category/sanitary-and-phytosanitary-sps-measures (The harmonised EAC SPS measures (under review) Section 2.8-(2.8.1), Section 3.7-(3.7.5), Section 3.7-(3.7.6) and Section 3.12) (accessed on 23 February 2021)
23. Government of Tanzania. *Livestock Extension Service Guidelines 2006.*, Government Press, Dar es Salaam, Tanzania, 27p, Available online: http://vetparaprofessional.blogspot.com/2012/08/livestock-extension-guideline.html (accessed on 23 February 2021)
24. World Trade Organization (WTO). Sanitary and Phyto-sanitary Measures Agreements., Available online: https://www.wto.org/english/tratop_e/sps_e/spsund_e.htm#:~:text=protecting%20domestic%20producers%3F-,The%20Agreement%20on%20the%20Application%20of%20Sanitary%20and%20Phytosanitary%20Measures,to%20set%20their%20own%20standards.&text=They%20should%20be%20applied%20only,or%20plant%20life%20or%20health. (accessed 23 March 2021)
25. Government of Tanzania. *The United Republic of Tanzania: The national action plan on antimicrobial resistance 2017-2022*, WHO, Dar es Salaam, Tanzania, 76p, Available online: https://www.who.int/publications/m/item/united-republic-of-tanzania-the-national-action-plan-on-antimicrobial-resistance (accessed on 23 February 2021)
26. World Health Organization. Global Action Plan on Antimicrobial Resistance. 2015. Available online: https://www.who.int/antimicrobial-resistance/global-action-plan/en/ (accessed on 23 February 2021).
27. Government of Tanzania and World Organisation for Animal Health. *OIE Performance of Veterinary Services (PVS) Evaluation Report 2008*, 188p, OIE, Paris, France, Available online: https://rr-africa.oie.int/wp-content/uploads/2019/11/tanzania-2016-fu.pdf (accessed on 23 February 2021)
28. Food and Agriculture Organization of the United Nations (FAO). *The FAO Action Plan on Antimicrobial Resistance 2016-2020*, FAO, Rome, Italy, 25p, Available online: http://www.fao.org/3/i5996e/i5996e.pdf (accessed on 23 February 2021)

29. Revolutionary Government of Zanzibar. *Zanzibar Livestock Policy;* 2011, Government Press, StoneTown, Zanzibar, 101p, Available online: http://www.tzdpg.or.tz/fileadmin/documents/dpg_internal/dpg_working_groups_clusters/cluster_1/agriculture/2._Ag_policies_and_strategies/Zanzibar_Ag_policies/2._2011_Zanzibar_Livestock_Policy_bk_edit.pdf (accessed on 23 February 2021)
30. Government of Tanzania. *Mwongozo wa Usajili wa Maduka ya Dawa Muhimu za Mifugo (in Swahili), 2015*, Government Press, Dar es Salaam, Tanzania, 32p, Available online: https://www.tanzania.go.tz/egov_uploads/documents/mwongozo_wa_usajili_wa_maduka_ya_dawa_muhimu_za_mifugo_sw.pdf (accessed on 23 February 2021)
31. Government of Tanzania. *Tanzania Pharmacy Act, 2011;* Act No.1 of 2011, Government Press, Dar es Salaam, Tanzania, 38p, Available online: https://www.pc.go.tz/files/THE%20PHARMACY%20ACT-2011.pdf (accessed on 23 February 2021)
32. Government of Tanzania. *Tanzania Water Supply and Sanitation Act, 2009;* Act No.12 of 2009, Government Press, Dar es Salaam, Tanzania, 31p, Available online: https://www.duwasa.go.tz/uploads/publications/en-1566284112-THE%20WATER%20SUPPLY%20%20AND%20SANITATION%20ACT%20NO%2012%20OF%202009.pdf (accessed on 23 February 2021)
33. Government of Tanzania. *Tanzania Public Health Act, 2009;* Act No.1 of 2009, Government Press, Dar es Salaam, Tanzania, 105p, Available online: https://www.tanzania.go.tz/egov_uploads/documents/The_Public_Health_Act,_2009_(Act_No_sw.pdf (accessed on 23 February 2021)
